# Real-world efficacy of bezlotoxumab for prevention of recurrent *Clostridium difficile* infection: a retrospective study of 46 patients in five university hospitals in Finland

**DOI:** 10.1007/s10096-019-03630-y

**Published:** 2019-07-29

**Authors:** Jarmo Oksi, A. Aalto, P. Säilä, T. Partanen, V.-J. Anttila, E. Mattila

**Affiliations:** 1 Department of Infectious Diseases, Turku University Hospital and Turku University, SH-rak 4.krs, PO Box 52, 20521 Turku, Finland; 2 Department of Medicine, Kuopio University Hospital, Kuopio, Finland; 3 Department of Infectious Diseases, Tampere University Hospital, Tampere, Finland; 4 Department of Internal Medicine, Oulu University Hospital, Oulu, Finland; 5 Department of Infectious Diseases, Helsinki University Central Hospital, Helsinki, Finland

**Keywords:** *Clostridium difficile* infection, Recurrence, Bezlotoxumab

## Abstract

Reports on real-world experience on efficacy of bezlotoxumab (BEZ) has been lacking thus far. We retrospectively studied the efficacy and safety of BEZ in preventing the recurrence of *Clostridium difficile* infection (CDI) in five university hospitals in Finland. Seventy-three percent of our 46 patients remained free of recurrence in the following 3 months and the performance remained as 71% effective also among immunocompromised patients. In severe CDI, BEZ prevented recurrence in 63% of cases. From our study patients, 78% had three or more known risk factors for recurrence of CDI. Eight of our patients were waiting for fecal microbiota transplantation but after stopping the antibiotics that were continued to prevent recurrence of CDI and after receiving BEZ, all remained free of recurrence and did not need the procedure. Success with BEZ as an adjunctive treatment in preventing recurrence of CDI in high-risk patients may be rated as high. Among a subgroup of our patients, those already evaluated to be in need of fecal microbiota transplantation, BEZ seems to be an alternative option.

## Introduction

Following a primary episode of *Clostridium difficile* infection (CDI), roughly 25% of patients treated with metronidazole or vancomycin will have a recurrent CDI (rCDI) in subsequent 3 months, most frequently in 2 to 3 weeks after cessation of the initial treatment regimen. After the first recurrence, the rate of rCDI increases to about 45% [1–4]. Fidaxomicin has shown to reduce the rate of rCDI to about 15–20%, even for those with a previous history of rCDI [5–7]. However, also all of the abovementioned antibiotics alter the normal gut microbiome and decrease colonization resistance against *C*. *difficile* [8]. Known risk factors for rCDI are age > 65 years, compromised immunity, severe CDI, prior CDI episode(s), and infection with hypervirulent ribotypes, hospitalization, inflammatory bowel disease, renal (or hepatic) impairment, antibiotic use during standard of care (SOC), antibiotic use after SOC in 3 months, and use of proton pump inhibitors [7, 9–14].

Thus far, fecal microbiota transplantation (FMT) has been shown to be the most effective treatment for rCDI [15, 16]. However, FMT is not available everywhere for different reasons, and all patients are not eligible for the procedure. Other treatment options are still needed.

Bezlotoxumab (BEZ) is a fully humanized monoclonal antibody against *C*. *difficile* toxin B and indicated for prevention of rCDI in at-risk patients [17, 18]. The efficacy and safety of BEZ were investigated among adults in global trials MODIFY I and MODIFY II in 2011–2015 [19]. In both studies, BEZ significantly reduced (*p* < 0.001) rCDI and had a favorable safety profile. Real-world performance of BEZ has been unknown since reports of new studies after launching of this immunological treatment given in combination with antibacterial drug treatment have been lacking thus far. The drug became commercially available for clinical use in February 2017. We now report our experience in Finnish university hospitals on the use of BEZ in first 46 patients with real-world comorbidities and risk for rCDI.

## Materials and methods

We investigated retrospectively the efficacy and safety of BEZ in an intent-to-treat setting in all (five) university hospitals in Finland (in Helsinki, Kuopio, Oulu, Tampere, and Turku) in 2017. The study was retrospective and therefore no uniform criteria on the degree of risk was set, but each clinician individually weighed the risks before making the decision to give BEZ in addition to standard of care (SOC). All hospitals used polymerase chain reaction (PCR) method to detect the gene for toxin production.

Renal impairment was defined with a background serum creatinine value more than 133 μl/l or 1.5 times the premorbid level or hemodialysis. Hepatic impairment was defined as having two or more of the following: an albumin level of 3.1 g/dl or lower, an alanine aminotransferase level at least 2 times the upper limit of the normal range, a total bilirubin level at least 1.3 times the upper limit of the normal range, or mild, moderate, or severe liver disease (as reported on the Charlson Index).

The study was retrospective and included the first 46 patients to receive BEZ in Finland in April–December 2017. Hospitalized patients as well as those who already were discharged from hospital were accepted for analysis. From patient records, we collected data on background diseases, immunosuppression, past CDIs, and severity (based on Zar score) [19] of the last CDI before the treatment with BEZ. Also, antibiotics other than for CDI were recorded during SOC and in 3 months after SOC. The dose of intravenous BEZ was 10 mg/kg in all but one patient weighing 149 kg, who got an infusion with 1000 mg only. BEZ was administered on 0–7 days after the initiation of SOC with the exception of those patients who were waiting for FMT. Subsequent data from 3 months after BEZ treatment was gathered from all patients. The information about rCDIs and other relevant information including antibacterial treatments given to the patients afterwards were collected from patient records and electronic pharmacy records.

## Results

In Helsinki University Hospital, BEZ was given to four hospitalized patients (inpatients, mean age 46 years) and to nine patients who came from home to get the infusion (outpatients, mean age 53 years). Table 1 presents different characteristics of the patients. Eight of the outpatients were waiting for fecal microbiota transplantation (FMT) after having had several (3 in five, 4 in two, and 2 in one patient) episodes of CDI and estimated to have a high risk of rCDI. All of these patients were on different dosages of oral vancomycin treatment before getting BEZ, but the treatment was discontinued on the day of the BEZ infusion. One patient with heart transplant was treated simultaneously with both FMT and BEZ. None of the eight patients waiting for FMT had a recurrent episode of CDI in 3 months after the treatment with BEZ—despite four of them were immunosuppressed. Therefore, the FMT was not needed anymore for these patients. From the four inpatients, three were immunocompromised and three had severe CDI (based on Zar score). One of these four had a rCDI. One outpatient was treated with BEZ twice—but still relapsed thereafter. One of the outpatients already had received FMT but after having rCDI 3 months later, he was successfully treated with BEZ.

**Table 1:** First 46 patients (pts) that received bezlotoxumab infusion in Finland in April–December 2017 (university hospitals)

	Helsinki	Kuopio	Oulu	Tampere	Turku
Total number of pts	13	7	5	7	14
Mean age (years)	56	74	71	59	69
Inpts	4	5	2	5	12
Immunocompromised pts	7	3	3	5	10
Severe CID, pts	5	2	2	4	4
Free of rCDI in 3 months, pts (%)	11 (85)	5 (71)	4 (80)	4 (57)	10 (71)

In Kuopio University Hospital, BEZ was given to seven high-risk patients (mean age 74 years, Table 1). Three of the patients were immunocompromised. Two of the seven patients were treated in ICU during the time of BEZ infusion. In their history, the patients had zero to four (median 2) previous CDI episodes. Two of the seven patients had a rCDI in the three following months after BEZ.

In Oulu University Hospital, BEZ was given to five patients (mean age 71 years, Table 1). One of them was a 97-year-old woman, living alone in her home, who had the seventh confirmed episode of CDI in 1 year and had already been treated with metronidazole for 14 days and with oral vancomycin altogether for 94 days before she got the BEZ infusion and a course of vancomycin for 28 days thereafter. In the following 3 months, she did not have a rCDI. Another patient was a 76-year-old patient with angioimmunoblastic T cell lymphoma, who had the fourth episode of CDI in 1 year. For the three previous episodes, the treatments were altogether 40 days oral metronidazole and 42 days oral vancomycin. The fourth episode without recurrence was treated with 14 days oral metronidazole and BEZ infusion. From the remaining three cases, one had the fifth episode of CDI but relapsed again after BEZ, and two had the third episode of CDI without recurrence after the BEZ.

In Tampere University Hospital, BEZ was given to seven patients (mean age 71 years, Table 1). Two of them were outpatients with a mild clinical picture of rCDI and came from home to receive the BEZ infusion. They did not get a relapse of CDI in 3 months. Five of the seven patients were hospitalized patients with severe infection in four of them and background immunosuppression also in four of the five patients. Three (all with severe CDI) of these five patients had a rCDI in 3 months despite BEZ treatment. One of these patients was diagnosed with hepatic carcinoma immediately after the BEZ treatment. The SOC in these three patients was vancomycin in all cases. Two of the five inpatients did not have a relapse although one of them, a hemodialysis patient, did have a long antibiotic treatment for spondylodiscitis after the BEZ treatment.

In Turku University Hospital, BEZ was given to fourteen patients, all of them inpatients (mean age 69 years, Table 1). Ten of the patients were immunocompromised and had several comorbidities. Three patients had acute leukemia or received allogenic hematologic stem cell transplantation (HSCT) in the previous few months. SOC for all of the hematologic patients included fidaxomicin. These patients were also neutropenic and continuously treated therefore with antibiotics after the BEZ infusion. Both of the HSCT patients did not have rCDI after BEZ infusion. Of the fourteen high-risk patients, five were treated with BEZ after the first CDI episode, while the number of CDI episodes was two in five patients, three in two patients, and four in two patients. Thus, the average number of CDI episodes in this very high-risk patients was 2.1. Two of the fourteen severely ill patients died during the following 3 months after the BEZ treatment—one due to end-stage cardiac disease (5 days after BEZ infusion), one due to graft-versus-host disease (GVHD) (1.5 months after BEZ). Ten of the fourteen (71%) patients in this very high-risk group did not have a rCDI in 3 months after the BEZ treatment. The last CDI episode in the group of fourteen patients was severe (based on Zar score) in four patients. None of these four patients relapsed in 3 months, but one of them died on GVHD, affecting also the intestine, already before 3 months had elapsed.

Altogether, 46 patients were treated, in addition to SOC, with BEZ infusion during April–December in 2017 in all university hospitals in Finland. Altogether, 32 (73%) of 44 patients did not have rCDI in 3 months after BEZ infusion. Two patients died before 3 months had elapsed after the BEZ infusion—and thus were not included to the group of “remaining free of rCDI.” One of the patients got the infusion twice but relapsed again after the second infusion. The mean age of all patients was 66 years (range 15–97 years). Twenty-two of the patients were female and 24 were men. The mean number of episodes of CDI was 2.74 (range 1–7). Seventeen patients were outpatients (eight of them waiting for FMT procedure) and 29 of the 46 patients were hospitalized at the time of BEZ infusion. Twenty-eight (61%) of the 46 patients were immunocompromised due to background comorbidity or immunosuppressive treatment. Twenty (71%) of the 28 immunocompromised patients did not have a rCDI after the treatment with BEZ. Seven of the 46 patients suffered from hepatic impairment and twelve from renal impairment. Based on the Zar score, 18 (39%) patients had severe CDI but the majority (10 of 16 patients, 63%) of them did not have a rCDI in 3 months after the treatment with BEZ (not eligible in two patients who died during the follow-up period). During SOC for the CDI episode, majority (28; 61%) of the high-risk patients had concomitant antibacterial treatment for another infection than CDI. During the following 3 months after the BEZ infusion, 19 (42%) of 45 patients (unknown for one patient) were treated for at least one antibacterial drug (other than those directed against *C*. *difficile*). The risk factors for rCDI and their frequency are listed in Table 2. Vancomycin was used in SOC partly or solely in 37 of 46 patients, metronidazole in nine, fidaxomicin in seven, and tigecycline in two. Adverse effects of BEZ were otherwise not found, but one patient experienced startling sensations after the infusion and one patient presented with fever in the following day after the infusion. The use of BEZ was regarded as safe by the investigators/treating physicians, since none of the possible adverse drug reactions had a probable causal relationship to the BEZ infusion.

**Table 2:** Frequency of the number of risk factors present in individual patients (*n* = 46) in the entire study population. Risk factors for rCDI (altogether 8 risk factors) recorded included the following: age > 65 years, compromised immunity, severe CDI, one or more previous CDI episodes, renal impairment, hepatic impairment, antibiotic use during SOC, antibiotic use after SOC in 3 months

Risk factors present	Number of patients, *n* = 46 (%)
0	0
1	3 (7)
2	7 (15)
3	7 (15)
4	13 (28)
5	14 (30)
6	2 (4)
7	0
8	0

## Discussion

In MODIFY I and II studies, BEZ demonstrated significant reductions in CDI recurrence compared with placebo (17% vs 28% in MODIFY I and 16% vs 26% in MODIFY II; *P* < 0.001) in adults receiving antibiotic treatment for primary CDI or rCDI [20]. In the MODIFY I and II, approximately 36% had a single risk factor, approximately 27% had 2 risk factors, and approximately 12% had ≥ 3 of previously identified five “high-risk” factors for rCDI: age ≥ 65 years, compromised immunity, severe CDI, prior CDI episode(s), and infection with ribotypes 027/078/244 [21]. Patients with more than or equal to three risk factors experienced the greatest reduction in CDI recurrence with BEZ (− 24.8% [range − 39.1%, − 9.3%]), suggesting a potential targeted patient population for the drug. One limitation of these data is that several of the risk factors are loosely defined, including immunocompromised patients, which was based on clinical criteria [21].

Patients with rCDIs often have extensive number of comorbidities and are at very high risk for a new recurrence. We therefore wanted to retrospectively investigate the real-world efficacy of BEZ in preventing rCDI. In our study, patients were often highly immunocompromised and 78% of all patients had three or more of eight listed (Table 2) risk factors for recurrence of CDI: 16 (35%) had 5–6 risk factors, 20 (43%) had 3–4 risk factors, and 10 (22%) had 1–2 risk factors. Definitions applied to severe CDI come from data from immunocompetent hosts and may not be applicable to the population of e.g. HSCT recipients. Severity of CDI in cancer patients may be underestimated for example due to the frequent presence of neutropenia [22]. Furthermore, patients with hematologic malignancies have lower creatinine levels at the time of CDI diagnosis compared with control patients. Therefore, CDI severity criteria based on white blood cell count and creatinine level may not be applicable to all patients [23].

Pooled clinical data from the MODIFY I and II studies demonstrated frequent use of concomitant antibiotics (37% in the BEZ arm vs 41% in the placebo arm) within the follow-up period (defined as the first day after the end of SOC treatment through onset of rCDI or day 90 following the infusion of study medication) [24, 25]. Respectively, 42% of patients in our study used concomitant antibiotics within follow-up period of 3 months. In our experience, the best results to prevent rCDI were achieved when BEZ was administered in a stable situation, as for example immediately before discharging from hospital or when all antibiotic treatments including SOC antibiotics were already discontinued (as was the case with those receiving BEZ when waiting for FMT). In this situation, intestinal microbiome gets time for recovery, contributing to healing. This treatment model is the same as with FMT, in which the aim is to avoid antibiotics after transplantation.

BEZ is generally well tolerated and reported adverse drug reactions did not differ significantly from placebo in the MODIFY I and II trials [20]. However, an unexplained increased risk of heart failure was noted (in 2.2% of BEZ and 0.9% of placebo arm) for patients with underlying congestive heart failure in phase III trials [17, 18]. In addition, 19.5% (23/118) and 12.5% (13/104) of this subgroup died, although the cause of death varied considerably. Heart failure and deaths were not infusion-related but occurred throughout the 12-week follow-up period. Heart failure is listed as warning and precaution in the US product insert and in these patients, BEZ should be reserved for use when benefit outweighs risk [17]. In our study, BEZ was well tolerated since we could not note any adverse drug-related reactions than possible infusion-related adverse reactions in two patients. However, one of our patients who had end-stage coronary artery disease and congestive heart failure died 5 days after BEZ infusion. The treating physician, however, regarded the succumbing as a natural event due to background comorbidity.

Clinical experience on BEZ has become from post hoc analyses of MODIFY I and II trials. The use of BEZ can reduce CDI-associated readmissions to hospitals especially in participants with high-risk prognostic factors [26]. The present real-world data on efficacy of BEZ in prevention of rCDI in very high-risk patients gives clinicians an estimate on its performance to compare this immunologic treatment with other strategies including FMT. However, the availability of FMT is limited—not all hospitals have resources to efficiently and timely use it. On the contrary, BEZ is an available treatment option in every hospital and may be offered also to patients that refuse FMT. Actually, the long-term safety of FMT is unclear. FMT is not a well-controlled procedure, whereas BEZ is a regulated medicine. There are also patients whose comorbid situation is not suitable (e.g., hematologic patients with neutropenia and/or allogenic transplants) for the use of FMT.

One limitation of our study is that we used only the toxin gene PCR for detection of CDI. It is likely that *C*. *difficile* will colonize patients for some time after the SOC. However, in our hospitals, the threshold to use the test is high—it is prohibited to use the test without a real clinical suspicion of CDI or the recurrence of it. Furthermore, the use of BEZ was always controlled by an infectious diseases specialist.

In conclusion, real-world experience on BEZ efficacy seems to be promising in our retrospective study of 46 patients in a university hospital setting in Finland. BEZ infusion as an adjunctive treatment to SOC was effective in the prevention of rCDI in 73% of patients and the performance remained as 71% effective also among immunocompromised patients. In severe CDI, 63% of cases remained free of rCDI in the following 3 months. From our study patients (*n* = 46), 78% had three or more known risk factors for recurrence of CDI. Therefore, success with BEZ together with SOC in preventing rCDI may be rated as high. Among a subgroup of our patients, those already evaluated to be in need of FMT, BEZ seems to be an alternative option.
